# Association of *SMC4* with prognosis and immune infiltration of sarcoma

**DOI:** 10.18632/aging.204503

**Published:** 2023-01-30

**Authors:** Guangyao Jiang, Junjie Chen, Yan Li, Jian Zhou, Wanchun Wang, Gen Wu, Yupeng Zhang

**Affiliations:** 1 Department of Orthopedics, People's Hospital of Pingchang County, Pingchang, Sichuan 636400, China; 2 Department of Orthopedics, Longhui People’s Hospital, Shaoyang, Hunan 422200, China; 3 Department of Orthopedics, The Second Xiangya Hospital, Central South University, Changsha, Hunan 410011, China; 4 Department of Orthopedics, The Fifth Affiliated Hospital, Southern Medical University, Guangzhou, Guangdong 510900, China; 5 Department of Spine Surgery, The Second Xiangya Hospital of Central South University, Changsha, Hunan 410011, China

**Keywords:** *SMC4*, sarcoma, prognosis, immune infiltrates, biomarker

## Abstract

Objective: This study was performed to explore the prognostic relevance of *structural maintenance of chromosomes 4* (*SMC4*) in pan-cancer and explore the association between *SMC4* and immune infiltration of sarcoma.

Results: Elevated expression of *SMC4* was detected in cancer tissues compared to normal tissue, which was confirmed in synovial sarcoma tissues with immunohistochemistry (IHC). Additionally, higher expression of *SMC4* was connected to worse outcomes of sarcoma, gastric cancer, breast cancer, liver cancer or ovarian cancer. Moreover, *SMC4* was positively connected to immune cell infiltrates in sarcoma. In addition, infiltrating immune cell markers including monocyte, TAM, M1 and M2 presented different *SMC4*-associated immune infiltration patterns.

Conclusion: The results from our study showed that *SMC4* was positively related to the prognosis and immunological status of sarcoma. *SMC4* could be a potential biomarker for prognosis and immune cell infiltrates in sarcoma.

Methods: Several databases including ONCOMINE, GEPIA, and Kaplan-Meier Plotter were adopted to explore the expression pattern of *SMC4* in sarcoma, which was confirmed by IHC. The GEPIA and TIMER datasets were adopted to investigate the associations between *SMC4* and prognosis in various cancers, especially in sarcoma.

## INTRODUCTION

Sarcoma is a type of tumor that originates from human mesenchymal cells [1]. Depending on where the tumor is located, sarcomas can be divided into more than 50 different types, such as osteosarcoma, liposarcoma and myxosarcoma. A report issued by the World Health Organization in the 2016 year showed that the incidence of sarcomas continues to increase [2]. In the United States, newly diagnosed sarcomas contribute to 19–21% of cancer-related deaths in childhood and adolescence [3]. Although most sarcomas are localized tumors that are typically treated with surgical resection and radiation therapy, the metastasis rate can be as high as 50% over the first five years after diagnosis (depending on the location, grade, and subtype). The five-year relative survival rate of patients with distant metastases is 16% due mainly to the limited efficacy of current systemic treatment programs [4].

The immune system can recognize and control the growth of cancer, but tumor cells can avoid recognition and elimination by the immune system to survive in the host. In previous studies, how cancer evades the immune system has been well-studied, which provides a helpful insight into preventing cancer immune evasion and eliminating cancer cells [5]. The diversity behind sarcomas has historically led to the development of effective new therapies that are slow and inefficient. Poor prognosis and clinical consequences for patients with sarcoma are still very common. A more in-depth understanding of the molecular pathology of specific sarcoma subtypes with modern immunotherapy technology holds the potential to inform the development of new treatments for sarcomas that are clinically effective [6]. To reach this goal, it is important to find new immunotherapy targets for sarcomas.

*SMC4* protein is encoded by the *SMC4* gene. *SMC4* and *SMC2* compose a heterodimer called condensing, which plays a crucial role in chromatin condensation and gene regulation [7, 8]. *SMC4* is a member of the *SMC* family genes and it is located in 3q25.33. According to a previous study, *SMC4* was highly conserved from bacteria to humans. Additionally, *SMC4* was associated with regulation of chromosome organization and dynamics [9]. Previous studies have also reported that expression of *SMC4* was significantly higher in lung adenocarcinoma, a type of sarcoma, and was significantly associated with a higher mortality rate [10]. Elevated expression of *SMC4* was discovered in liver cancer and colon cancer and can promote their growth [11]. However, the exact role and mechanism of *SMC4* in promoting the progression of sarcomas remain unclear.

To this end, we have conducted this study with the aim to show the prognostic significance of *SMC4* in sarcoma and its interaction with infiltrating immune cells. The expression level of *SMC4* in sarcoma was detected using ONCOMINE and TIMER datasets, and then used IHC to confirm the elevated expression of *SMC4*. The prognostic significance of *SMC4* in pan-cancer was revealed by GEPIA and Kaplan-Meier Plotter datasets. GEPIA and TIMER databases were used to discover the relationships of *SMC4* with immune cell infiltration in sarcoma.

## RESULTS

### mRNA expression level of *SMC4* in pan-cancer

**Figure 1:**
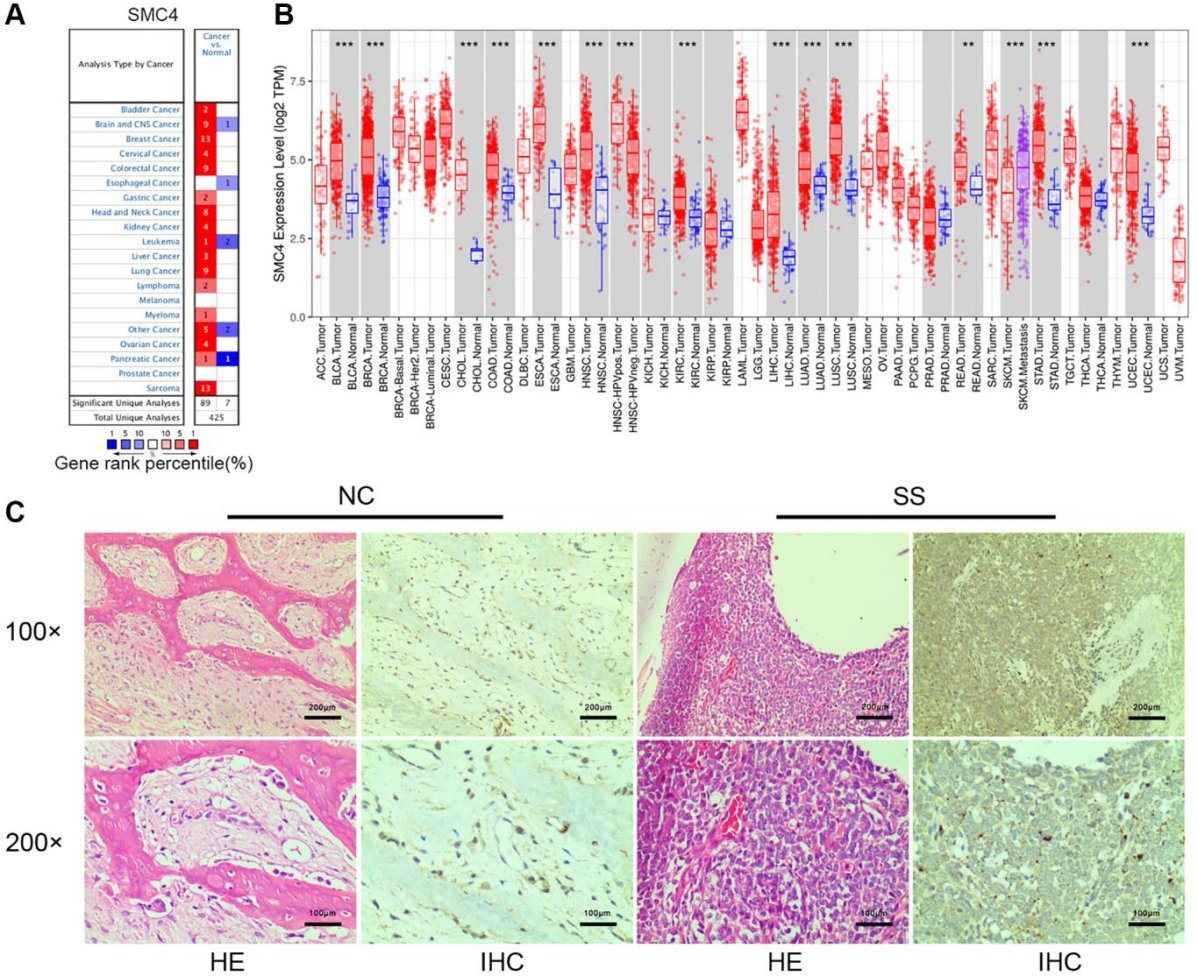
**Expression and prognostic value of *SMC4* in various cancers.** (**A**) The differential expression of *SMC4* in multiple cancer tissues compared to normal tissues using the Oncomine database; (**B**) *SMC4* expression in various cancers from TCGA database. Note: ^*^p < 0.05, ^**^p < 0.01, ^***^p < 0.001; (**C**) The Expression of *SMC4* in synovial sarcoma tissue. Abbreviations: NC: normal control; SS: synovial sarcoma.

We analyzed the expression level of *SMC4* mRNA in various cancers using Oncomine dataset. *SMC4* was highly expressed in tumor samples including bladder cancer, kidney cancer, brain and CNS cancer, head and neck cancer, liver cancer, breast cancer, cervical cancer, colorectal cancer, gastric cancer, myeloma, lung cancer, lymphoma, ovarian cancer and sarcoma compared to normal tissue samples (Figure 1A and Supplementary Table 1). Then TCGA RNA-seq data in TIMER database was analyzed. The mRNA expression of *SMC4* was significantly higher in SKCM, LUAD, BLCA, BRCA, READ, CHOL, STAD, COAD, UCEC, ESCA, HNSC, KIRC, LIHC, LUSC and SARC (Figure 1B). Furthermore, IHC was used to explore the expression of *SMC4* in sarcoma tissue. We found that *SMC4* was overexpressed in synovial sarcoma (Figure 1C).

### Prognostic value of *SMC4* in tumors

**Figure 2:**
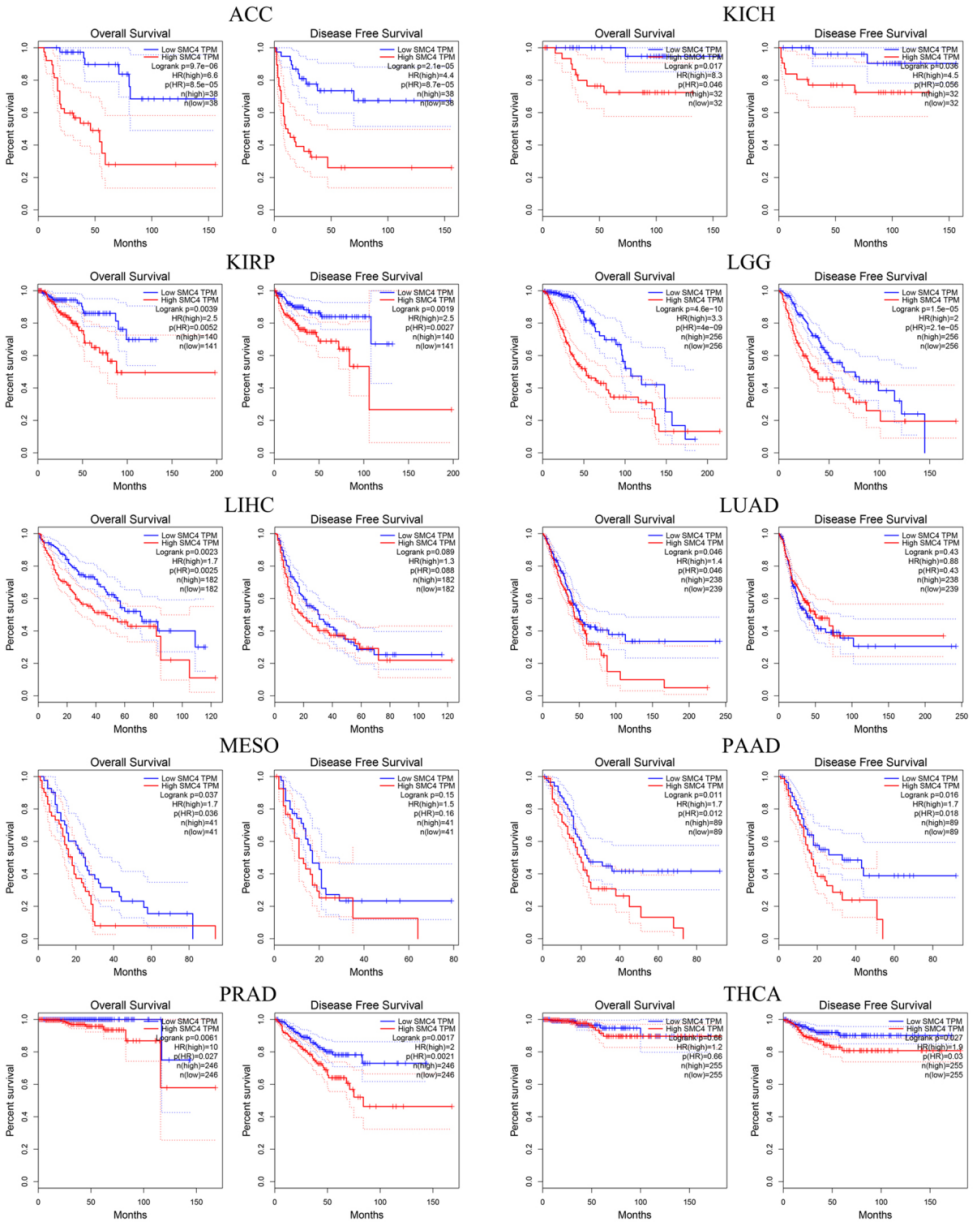
Prognostic significance of *SMC4* in different types of human cancers using GEPIA database.

The GEPIA database was adopted to analyze the prognostic value of *SMC4* in multiple cancers. *SMC4* was observed to be significantly related to poorer overall survival (OS) and disease-free survival (DFS) in ACC, KICH, KIRP, LGG, PAAD and PRAD, poorer OS in LIHC, LUAD and MESO and poorer disease-free survival post-THCA (Figure 2).

**Figure 3:**
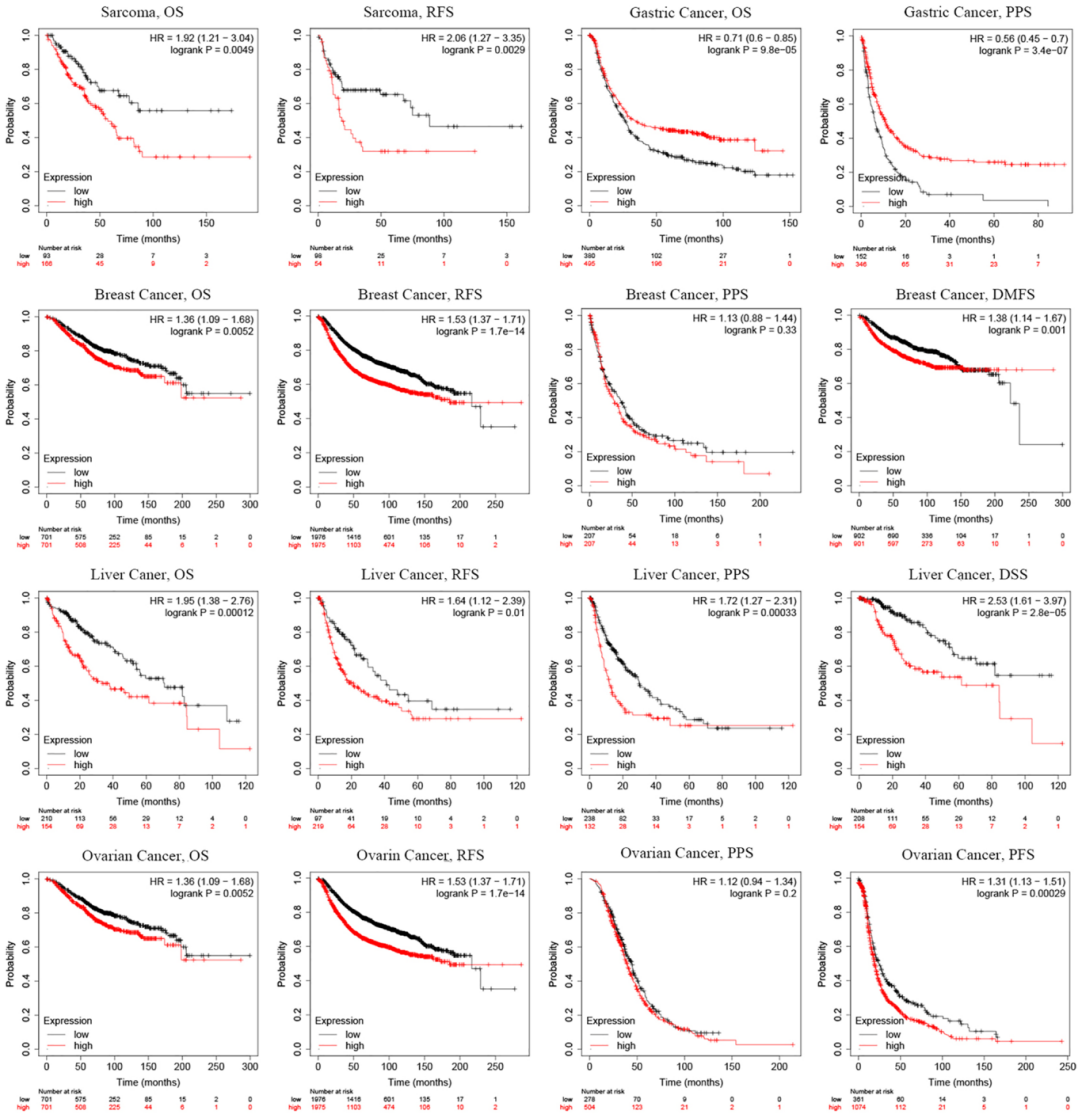
**Prognostic significance of *SMC4* in different types of human cancers using the Kaplan-Meier plotter database.** Abbreviations: OS: overall survival; PFS: progression-free survival; RFS: relapse-free survival; DSS: disease-specific survival; DMFS: distant metastasis-free survival; PPS: post progression survival.

Additionally, the prognostic significance of *SMC4* in pan-cancer was detected using the Kaplan-Meier Plotter database. Higher expressed *SMC4* related to the poorer prognosis of sarcoma (OS: HR = 1.92, *P* = 0.0049; RFS: HR = 2.09, *P* = 0.0029), breast cancer (OS: HR = 1.36, *P* = 0.0052; RFS: HR = 1.53, *P* = 1.7e-14; DMFS: HR = 1.38, *P* = 0.001), liver cancer (OS: HR = 1.95, *P* = 0.00012; RFS: HR = 1.64, *P* = 0.01; PPS: HR = 1.72, *P* = 0.00033; DSS: HR = 2.53, *P* = 2.8e-05) and ovarian cancer (OS: HR = 1.36, *P* = 0.0052; RFS: HR = 1.53, *P* = 1.7e-14; PFS: HR=1.31, *P* = 0.00029). In contrast, a higher expression level of *SMC4* predicted a better prognosis of Gastric Cancer (OS: HR = 0.71, *P* = 9.8e-05; PPS: HR = 0.56, *P* = 3.4e-07) (Figure 3). These results indicated that *SMC4* might be a prognostic biomarker in these cancers.

### Association between *SMC4* and immune cell infiltration of sarcoma

**Figure 4:**
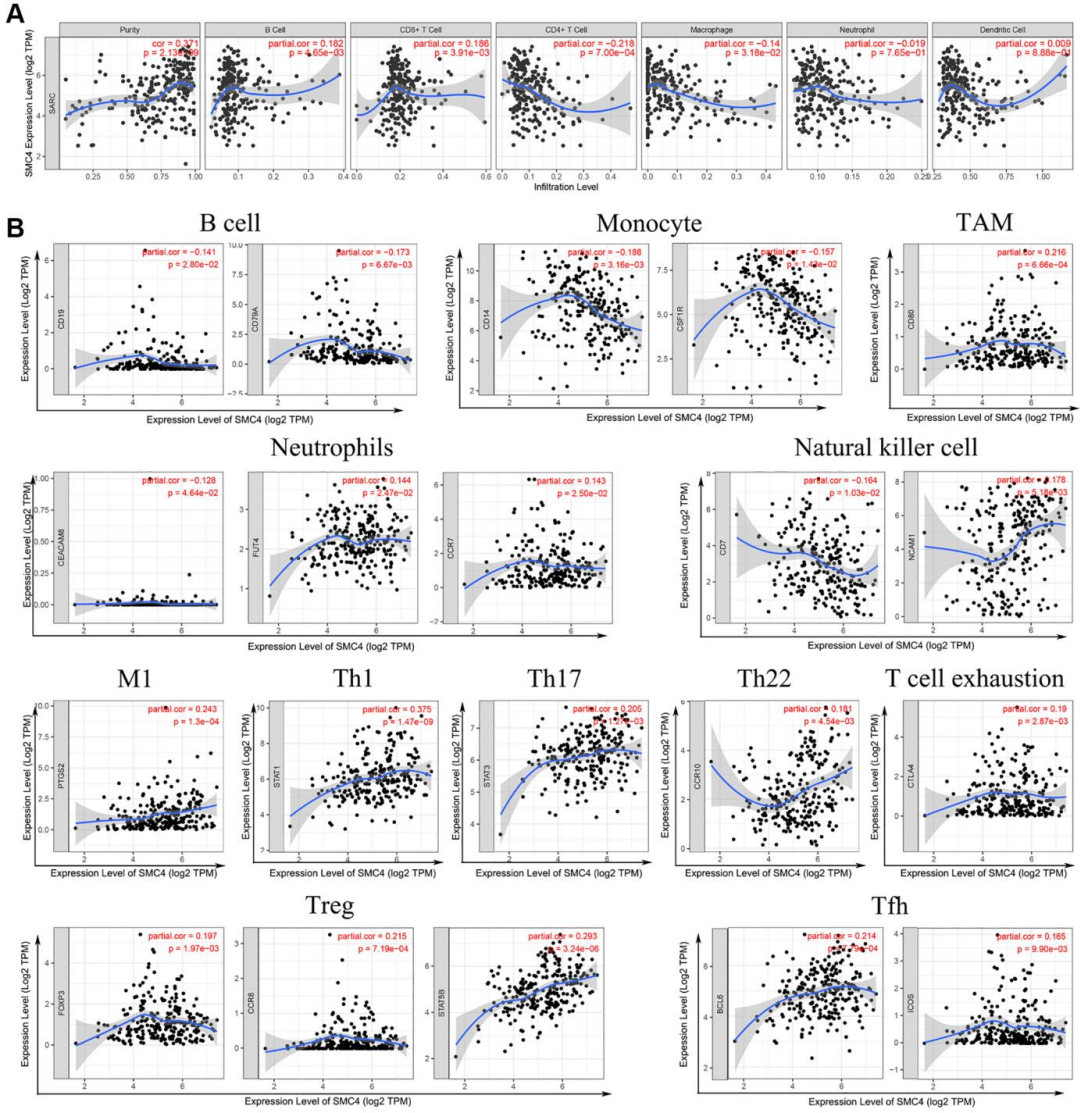
Correlation between *SMC4* expression and immune infiltration of sarcoma (**A**) Data form TIMER database; (**B**) *SMC4* associated immune infiltration patterns by infiltrating immune cells markers.

The association between *SMC4* and immune cell infiltration in 39 cancer types, including sarcoma was analyzed using the TIMER dataset. The expression level of *SMC4* was connected to the infiltration level of dendritic cells, CD4+ T cells, neutrophils, B cells, CD8+ T cells (Figure 4A) and macrophages in 22, 24, 20, 24, 26, and 23 tumors, respectively (Table 1). For the sarcomas, the expression level of *SMC4* was related to the infiltration levels of B cells (r = 0.182, *P* = 4.65e-03) and CD8+T cells (r = 0.186, *P* = 3.91e-03), negatively related to the infiltration levels of CD4+ T cell (r = −0.218, *P* = 7.00e-04) and macrophage (r = −0.14, *P* = 3.18e-02) (Figure 4A).

**Table 1:** Correlation between SMC4 expression and immune infiltration of pan-cancer using TIMER database.

**Cancer type**	**Purity**		**B cell**		**CD8+ T Cell**		**CD4+ T Cell**		**Macrophage**		**Neutrophil**		**Dendritic Cell**	
	**Cor**	***p***	**Cor**	***p***	**Cor**	***p***	**Cor**	***p***	**Cor**	***p***	**Cor**	***p***	**Cor**	***p***
ACC	0.368	1.25e-03	0.296	1.11e-02	0.138	2.44e-01	0.148	2.10e-01	0.063	5.95e-01	0.239	4.21e-02	0.302	9.48e-03
BLCA	0.106	4.12e-02	0.032	5.43e-01	0.291	1.48e-08	0.066	2.10e-01	0.075	1.51e-01	0.267	2.54e-07	0.317	6.09e-10
BRCA	0.181	8.56e-09	0.199	3.23e-10	0.258	2.37e-16	0.128	6.70e-05	0.078	1.40e-02	0.27	2.50e-17	0.198	7.63e-10
BRCA-Basal	0.026	7.70e-01	0.203	2.36e-02	0.206	2.20e-02	0.266	3.10e-03	0.071	4.29e-01	0.353	1.57e-04	0.336	2.52e-04
BRCA-Her2	0.037	7.79e-01	−0.24	6.92e-02	−0.067	6.23e-01	0.219	9.87e-02	−0.003	9.82e-01	0.175	1.90e-01	0.045	7.41e-01
BRCA-Luminal	0.26	6.44e-10	0.145	7.22e-04	0.234	4.04e-08	0.13	2.66e-03	0.116	6.78e-03	0.224	1.78e-07	0.163	1.57e-04
CESC	0.107	7.38e-02	−0.059	3.31e-01	0.054	3.69e-01	0.09	1.37e-01	−0.141	1.91e-02	0.104	8.39e-02	0.052	3.90e-01
CHOL	−0.311	6.52e-02	−0.038	8.31e-01	0.096	5.83e-01	−0.004	9.83e-01	0.123	4.81e-01	0.307	7.30e-02	0.015	9.32e-01
COAD	0.063	2.08e-01	0.282	7.71e-09	0.281	8.23e-09	0.165	8.68e-04	0.186	1.76e-04	0.259	1.41e-07	0.194	9.07e-05
DLBC	−0.064	6.87e-01	0.596	9.01e-03	−0.216	3.47e-01	−0.161	4.86e-01	0.051	8.27e-01	0.09	6.97e-01	0.158	4.95e-01
ESCA	0.212	4.18e-03	0.145	5.33e-02	0.027	7.22e-01	−0.115	1.26e-01	0.129	8.46e-02	−0.022	7.65e-01	−0.127	9.05e-02
GBM	0.402	1.07e-17	−0.05	3.11e-01	0.02	6.85e-01	−0.125	1.05e-02	0.023	6.41e-01	−0.021	6.70e-01	0.15	2.15e-03
HNSC	0.213	1.80e-06	0.114	1.27e-02	0.107	1.94e-02	0.322	4.70e-13	0.16	4.18e-04	0.213	2.50e-06	0.225	6.02e-07
HNSC-HPVpos	0.142	1.82e-01	0.221	5.06e-02	0.269	1.75e-02	0.311	4.39e-03	0.033	7.64e-01	0.273	1.16e-02	0.213	5.28e-02
HNSC-HPVneg	0.168	7.48e-04	−0.001	9.80e-01	0.002	9.72e-01	0.306	5.49e-10	0.158	1.64e-03	0.174	5.32e-04	0.184	2.31e-04
KICH	0.222	7.28e-02	0.144	2.52e-01	0.242	5.17e-02	−0.118	3.48e-01	0.251	4.37e-02	−0.091	4.72e-01	0.182	1.47e-01
KIRC	−0.16	5.63e-04	0.303	3.31e-11	0.339	3.32e-13	0.388	5.46e-18	0.332	5.53e-13	0.539	8.33e-36	0.469	3.16e-26
KIRP	0.047	4.55e-01	0.101	1.07e-01	0.046	4.57e-01	0.075	2.31e-01	−0.159	1.19e-02	0.234	1.46e-04	0.137	2.88e-02
LGG	0.073	1.09e-01	0.361	3.86e-16	0.392	5.84e-19	0.241	9.79e-08	0.385	4.01e-18	0.317	1.55e-12	0.346	7.57e-15
LIHC	0.127	1.79e-02	0.398	1.67e-14	0.297	2.15e-08	0.441	8.30e-18	0.494	2.16e-22	0.475	8.18e-21	0.44	1.51e-17
LUAD	0.056	2.11e-01	−0.01	8.31e-01	0.204	5.95e-06	0.052	2.52e-01	0.017	7.14e-01	0.222	8.05e-07	0.144	1.38e-03
LUSC	0.28	4.98e-10	−0.025	5.87e-01	−0.038	4.08e-01	−0.059	2.01e-01	−0.121	8.12e-03	−0.092	4.41e-02	−0.086	6.28e-02
MESO	−0.202	6.19e-02	0.337	1.72e-03	0.255	1.94e-02	−0.028	8.00e-01	0.316	3.43e-03	−0.087	4.32e-01	0.296	6.32e-03
OV	−0.058	2.06e-01	0.099	3.08e-02	−0.039	3.91e-01	0.144	1.60e-03	0.109	1.69e-02	0.125	6.05e-03	0.085	6.29e-02
PAAD	−0.032	6.75e-01	0.305	4.86e-05	0.427	5.53e-09	−0.172	2.56e-02	0.3	6.82e-05	0.294	9.30e-05	0.418	1.31e-08
PCPG	0.14	6.99e-02	0.275	3.25e-04	0.093	2.33e-01	0.008	9.14e-01	0.158	4.23e-02	0.196	1.10e-02	0.003	9.69e-01
PRAD	−0.007	8.86e-01	0.515	2.87e-29	0.469	3.95e-24	0.233	1.73e-06	0.353	1.22e-13	0.467	8.22e-24	0.458	6.13e-23
READ	0.004	9.64e-01	0.193	2.25e-02	0.351	2.23e-05	−0.034	6.94e-01	0.079	3.55e-01	0.295	4.50e-04	0.064	4.57e-01
SKCM	0.07	1.34e-01	0.111	1.86e-02	0.377	3.17e-16	0.104	2.80e-02	0.219	2.63e-06	0.535	8.47e-35	0.263	1.74e-08
SKCM-Primary	0.236	1.64e-02	0.037	7.14e-01	0.455	1.76e-06	−0.127	2.04e-01	0.202	4.26e-02	0.582	2.22e-10	0.191	5.62e-02
SKCM-Metastasis	0.026	6.26e-01	0.034	5.27e-01	0.29	6.53e-08	0.086	1.13e-01	0.143	7.28e-03	0.454	3.14e-19	0.183	6.33e-04
STAD	0.105	4.08e-02	−0.017	7.44e-01	−0.129	1.31e-02	−0.103	4.81e-02	−0.265	2.18e-07	−0.046	3.76e-01	−0.124	1.69e-02
TGCT	−0.054	5.17e-01	0.226	5.90e-03	0.413	2.03e-07	−0.059	4.81e-01	0.009	9.18e-01	0.053	5.23e-01	0.278	6.80e-04
THCA	0.018	6.85e-01	0.627	5.06e-54	−0.153	6.93e-04	0.496	1.18e-31	0.544	6.63e-39	0.404	1.54e-20	0.452	8.61e-26
THYM	−0.123	1.89e-01	0.725	7.46e-20	0.559	1.01e-10	0.569	7.49e-11	0.588	6.26e-12	−0.03	7.55e-01	0.71	9.40e-19
UCEC	0.048	4.07e-01	−0.115	5.03e-02	0.098	9.80e-02	−0.107	6.84e-02	−0.043	4.66e-01	0.295	2.63e-07	−0.051	3.89e-01
UCS	0.151	2.76e-01	0.005	9.72e-01	0.085	5.44e-01	−0.157	2.62e-01	0.261	5.90e-02	−0.144	3.03e-01	−0.007	9.62e-01
UVM	−0.003	9.77e-01	0.406	3.05e-04	0.284	1.25e-02	−0.291	1.07e-02	−0.139	2.75e-01	0.257	2.41e-02	−0.182	1.21e-01

### *SMC4* expression and immune markers

The associations between *SMC4* and markers of various immune cells in pan-cancer were detected using GEPIA database. We found that the expression level of *SMC4* in tumor tissues is associated with the expression level of gene markers of tumor-infiltration immune cells including exhausted T cells, NK cells, Tfh, Treg, monocyte, TAM, B cell, T cell and neutrophils (Table 2). Additionally, we further studied the association of *SMC4* with tumor-infiltrating immune cells in sarcoma tissues. *SMC4* expression had a significant correlation with the expression of markers of B cell (CD19 and CD79A), monocyte (CD14, CSF1R and CD86), neutrophils (CEACAM8, FUT4 and CCR7), natural killer cells (CD7 and NCAM1), TAM (CD80), M1 macrophage (PTGS2), and various subtypes of T cells including Th1 (STAT1), Th17 (STAT3), Th22 (CCR10), exhausted T cells (CTLA4), Treg (FOXP3, CCR8 and STAT5B), and Tfh (BCL6 and ICOS) (Table 3 and Figure 4B). As indicated in Figure 1, *SMC4* was highly expressed in sarcoma tissue. These results confirm that in sarcoma patients, the expression level of *SMC4* is significantly related to the infiltration level of immune cells.

**Table 2:** Correlations between SMC4 and genes markers of immune cells in GEPIA.

**Description**	**Gene markers**	**Tumor**		**Normal**	
		**R**	***P***	**R**	***P***
B cell	CD19	0.027	^**^	0.11	^**^
	CD79A	0.02	0.054	0.1	^**^
T cell (general)	CD2	0.1	^***^	0.56	^***^
	CD3D	0.044	^***^	0.5	^***^
	CD3E	0.084	^***^	0.53	^***^
Monocyte	CD14	−0.086	^***^	-0.22	^***^
	CSF1R	0.0086	0.4	0.22	^***^
	CD86	0.17	^***^	0.31	^***^
TAM	CD80	0.17	^***^	0.3	^***^
	CCL2	−0.049	^***^	0.018	0.63
	CD68	0.03	^**^	0.25	^***^
	IL10	0.067	^***^	0.14	^***^
Neutrophils	CEACAM8	0.14	^***^	0.1	^**^
	FUT4	0.35	^***^	0.24	^***^
	CCR7	0.044	^***^	0.33	^***^
Natural killer cell	CD7	0.1	^***^	0.44	^***^
	NCAM1	−0.15	^***^	0.015	0.68
Tfh	BCL6	0.16	^***^	0.3	^***^
	ICOS	0.2	^***^	0.43	^***^
	CXCR5	0.036	^***^	0.22	^***^
Treg	FOXP3	0.15	^***^	0.25	^***^
	CCR8	0.17	^***^	0.46	^***^
	STAT5B	0.23	^***^	0.2	^***^
Exhausted T cell	CTLA4	0.11	^***^	0.26	^***^

^*^P < 0.05; ^**^P < 0.01; ^***^P < 0.001.

**Table 3:** Correlations between SMC4 and gene markers of immune cells in TIMER.

**Description**	**Gene markers**	**None**		**Purity**	
		**Cor**	***p***	**Cor**	***p***
CD8+ T cell	CD8A	−0.048	0.444	0.099	0.121
	CD8B	0.052	0.262	0.015	0.821
T cell (general)	CD2	−0.105	0.092	0.065	0.312
	CD3D	−0.173	^**^	−0.003	0.962
	CD3E	−0.105	0.090	0.062	0.339
B cell	CD19	0.071	0.536	−0.141	^*^
	CD79A	−0.294	^***^	−0.173	^**^
Monocyte	CD14	−0.347	^***^	−0.188	^**^
	CSF1R	−0.324	^***^	−0.157	^*^
	CD86	−0.194	^**^	0.007	0.910
TAM	CD80	0.057	0.358	0.216	^***^
	CCL2	−0.116	0.062	−0.014	0.829
	CD68	−0.279	^***^	−0.099	0.125
	IL10	−0.209	^***^	−0.02	0.755
M1 Macrophage	NOS2	−0.106	0.089	−0.064	0.323
	IRF5	−0.213	^***^	−0.033	0.605
	PTGS2	0.319	^***^	0.243	^***^
M2 Macrophage	CD163	−0.21	^***^	−0.009	0.886
	VSIG4	−0.248	^***^	−0.064	0.321
	MS4A4A	−0.253	^***^	−0.058	0.365
	ARG1	0.075	0.225	0.053	0.411
	MRC1	−0.203	^**^	−0.045	0.484
Neutrophils	CEACAM8	−0.106	0.088	−0.128	^*^
	CD11b	−0.24	^***^	−0.072	0.264
	FUT4	0.098	0.116	0.144	^*^
	CCR7	0.036	0.563	0.143	^*^
Natural killer cell	CD7	−0.296	^***^	−0.164	^*^
	NCAM1	0.296	^***^	0.178	^**^
Th1	T-bet	−0.118	0.058	0.011	0.863
	STAT1	0.285	^***^	0.375	^***^
	STAT4	−0.213	^***^	−0.042	0.510
Th2	GATA3	−0.108	0.083	0.018	0.774
	STAT5A	−0.071	0.257	0.02	0.757
	STAT6	0.108	0.082	0.041	0.527
	CCR3	−0.078	0.210	−0.014	0.831
	IL13	0.022	0.727	0.071	0.268
Th17	IL21R	−0.085	0.171	0.098	0.128
	IL23R	0.081	0.196	0.111	0.084
	STAT3	0.257	^***^	0.205	^**^
Th22	CCR10	0.261	^***^	0.181	^**^
	AHR	−0.018	0.775	0.066	0.304
Tfh	BCL6	0.236	^***^	0.214	^***^
	CXCR5	−0.09	0.150	−0.032	0.616
	ICOS	0.001	0.981	0.165	^**^
Treg	FOXP3	0.055	0.374	0.197	^**^
	CCR8	0.127	^*^	0.215	^***^
	STAT5B	0.397	^***^	0.293	^***^
T cell exhaustion	CTLA4	0.013	0.833	0.19	^**^
	LAG3	−0.064	0.305	0.054	0.397
	GZMB	−0.165	^**^	−0.014	0.823
	PDCD-1	−0.191	^**^	−0.026	0.683
	HAVCR2	−0.229	^***^	−0.025	0.693

### Relationship between the prognostic significance of *SMC4* in sarcoma and immune cells

*SMC4* was found to be significantly related to poorer OS in sarcoma patients with reduced eosinophils, CD8+ T-cells, CD4+ memory T cells, B cells, basophils, natural killer T-cells, regulatory T-cells and type 2 T-helper cells. Meanwhile, we observed that expression of *SMC4* was positively related to sarcoma patients with enriched basophils, type 1 T-helper cells, macrophages, mesenchymal stem cells, type 2 T-helper cells and regulatory T-cells (Table 4).

**Table 4:** Correlation of SMC4 mRNA expression and overall survival rate in sarcoma with different immune cells by Kaplan-Meier plotter.

**Immune cells**	**Enriched**			**Decreased**		
	***N***	**Hazard ratio**	***P*-value**	***N***	**Hazard ratio**	***P*-value**
Basophils	155	1.84 (1.03−3.29)	0.036^*^	103	2.76 (1.39−5.49)	0.0026^*^
B-cells	64	2.17 (0.94−5)	0.063	194	2.01 (1.14−3.54)	0.014^*^
CD4+ memory T-cells	84	1.86 (0.7−4.94)	0.2	174	2.24 (1.27−3.97)	0.0044^*^
CD8+ T-cells	122	1.71 (0.93−3.13)	0.08	136	2.37 (1.19−4.7)	0.011^*^
Eosinophils	75	2.22 (0.66−7.41)	0.19	183	2.12 (1.26−3.55)	0.0037^*^
Macrophages	145	1.95 (1.09−3.49)	0.022^*^	113	1.92 (0.98−3.76)	0.053
Mesenchymal stem cells	172	2.28 (1.41−3.7)	0.00054^*^	86	1.62 (0.79−3.35)	0.18
Natural killer T-cells	166	1.77 (0.89−3.52)	0.1	92	2.51 (1.23−5.13)	0.0089^*^
Regulatory T-cells	68	2.48 (1.11−5.54)	0.022^*^	190	195 (1.12−3.4)	0.016^*^
Type 1 T-helper cells	122	2.61 (1.36−5)	0.0027^*^	136	1.47 (0.76−2.84)	0.25
Type 2 T-helper cells	217	1.78 (1.06−2.97)	0.026^*^	41	3.1 (1.03−9.31)	0.034^*^

^*^P < 0.05.

### Gene expression profiling between the *SMC4* high group and *SMC4* low group

**Figure 5:**
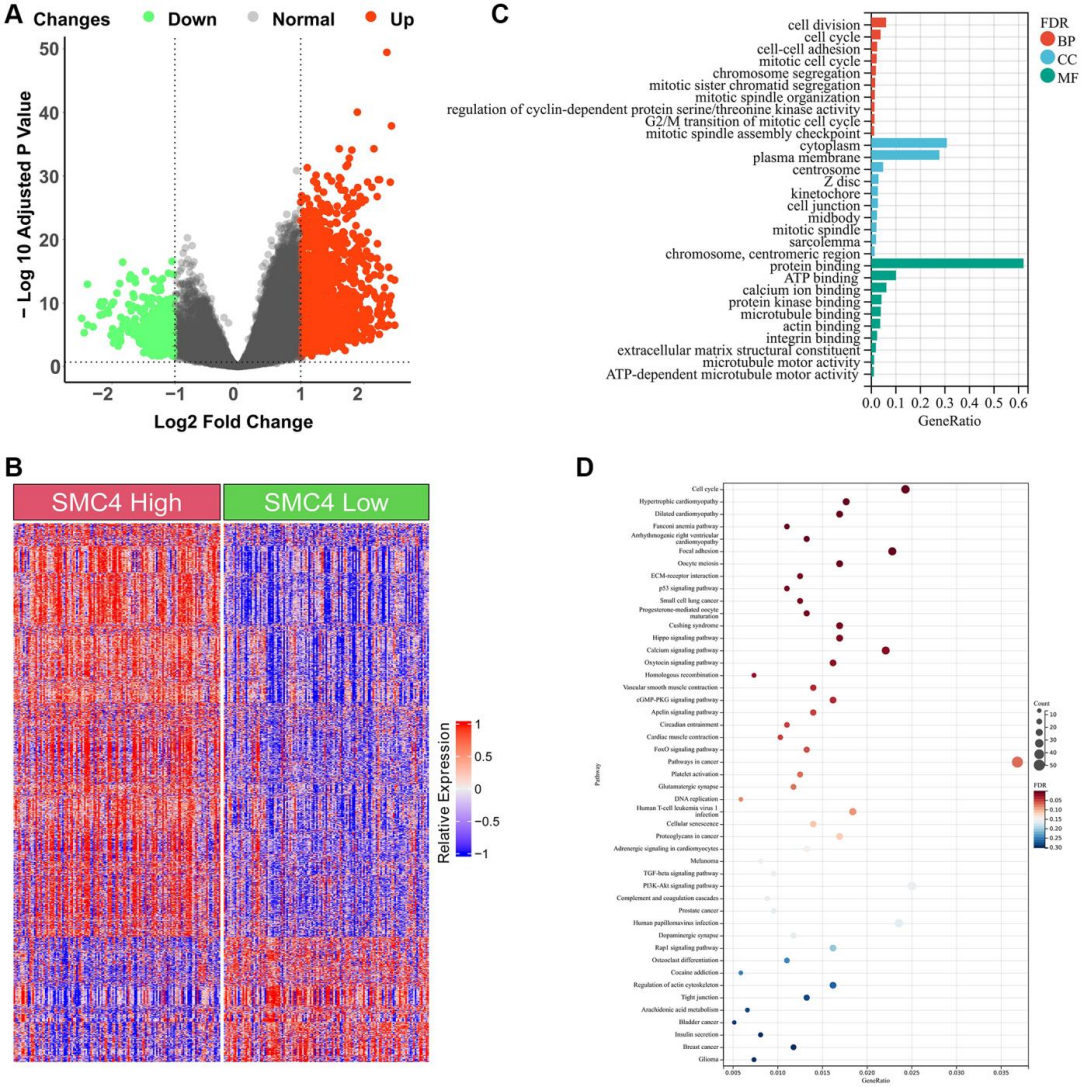
**Enrichment analysis of DEGs between *SMC4* high group and *SMC4* low group.** (**A**) Volcano plot of DEGs; (**B**) Heatmap of DEGs; (**C**) GO analysis; (**D**) KEGG analysis.

Many pathways may be changed between the *SMC4* high group and the *SMC4* low group in sarcomas. Therefore, we analyzed the DEGs between the *SMC4* high group and the *SMC4* low group using data on sarcoma from TCGA. GO and KEGG analyses were performed. As shown in Figure 5, a total of 1613 DEGs including 1239 up-regulated genes and 374 down-regulated genes were detected (Figure 5A, 5B). GO analysis indicated that DEGs between the *SMC4* high group and the *SMC4* low group were mainly enriched in cell division for BP, cytoplasm for CC and protein binding for MF (Figure 5C). Additionally, we observed that DEGs between the *SMC4* high group and the *SMC4* low group were mainly enriched in pathways in cancer, PI3K-Akt signaling pathway and cell cycle pathway from KEGG analysis (Figure 5D).

## DISCUSSION

*SMC4* was an essential component of condensing. It promotes the condensation of chromatin during the mitosis of eukaryotic cells [12]. Yan et al. reported that *SMC4* was related to a poor prognosis and immune infiltration levels of hepatocellular carcinoma [13]. He et al. found that overexpression of *SMC4* facilitated the development of cervical cancer cells [14]. Moreover, Zhu et al. observed that *SMC4* expression was connected to a poor prognosis in ovarian cancer patients [15]. Previous studies have shown that *SMC4* was an active regulator of inflammatory innate response and highly expressed in sarcomas and some other cancers [7, 12, 16–19], but it is still unknown whether the elevated expression of *SMC4* in tumor tissues is related to the infiltration of immune cells.

The expression level of *SMC4* mRNA in different tumor tissues and normal tissues was detected using the ONCOMINE database and TIMER database. The results showed that in a variety of tumor tissues, the expression level of *SMC4* was significantly up-regulated. The results of our prognostic analysis in the Kaplan-Meier Plotter database show that the up-regulation of *SMC4* expression levels is significantly related to the poor prognosis of patients with sarcoma, breast cancer, liver cancer, or ovarian cancer. In addition, the analysis of the GEPIA database showed that the higher expression of *SMC4* was associated with poorer prognosis of more tumors, including THCA, PRAD, PAAD, ACC, LGG, MESO, LUAD, KICH, KIRP and LIHC. Elevated expression of *SMC4* is related to the poor outcome of tumor patients. In patients with sarcoma, the relationship between *SMC4* and poor OS and RFS indicates that *SMC4* is a potential prognostic biomarker for sarcoma.

Our research also indicated that *SMC4* was connected to the degree of immune cell infiltration in a variety of tumors, including sarcoma. In sarcoma, the expression of *SMC4* was correlated with the infiltration of B cells and CD8+ T cells but negatively correlated with the immune infiltration of CD4+ T cells and macrophages. Under different immune cell infiltration conditions, *SMC4* has different prognostic significance for sarcoma. *SMC4* may affect the prognosis of patients with sarcoma by regulating tumor immune function.

In the early stage of tumorigenesis, the immune system activates T cells and macrophages to attack tumor cells to prevent the development of cancer. However, after this stage, immune TME will turn to support cancer cells and promote tumor progression, while suppressing immune cell-mediated cytotoxicity [20]. Specifically, in sarcoma, the expression level of *SMC4* is significantly correlated with monocyte markers *CD14* and *CSF1R*, TAM marker *CD80*, and M1 macrophage marker *PTGS2*. This indicates that the expression of *SMC4* plays an important role in regulating the infiltration and activity of TAM. In the past, researchers have confirmed the tumor-promoting effect of TAM in sarcoma, and there is accumulating evidence showing the anti-tumor effect of TAM-targeted therapy [21]. By analyzing the relationship between the expression of *SMC4* and the markers of helper T cells, we found that the expression level of *SMC4* is related to Th1 (*STAT1*), Th17 (*STAT3*), Th22 (*CCR10*), Treg (*FOXP3, CCR8* and *STAT5B*) and Tfh (*BCL6* and *ICOS*) and other helper T cell markers are related to the expression. This suggests that *SMC4* plays a role in regulating the tumor immune infiltration of T helper cells. In addition, *SMC4* is positively related to the expression of depleted T cell marker *CTLA4*, and *CTLA4* is a key inhibitory immune checkpoint protein [22], which suggests that the high expression of *SMC4* may inhibit the function of T cells and evade the immune response, thus promoting the development of sarcoma [23]. This pathway has also been reported in many other tumors [24–26]. Our study also shows that the lower expression of *SMC4* is related to a poorer prognosis of sarcoma and more infiltration of various immune cells, including B cells, neutrophils monocytes, NK cells and macrophages. The expression of *SMC4* is also related to the infiltration of Th, Treg and depleted T cells. These data indicate that *SMC4* could be a potential independent biomarker of sarcoma prognosis and tumor immune status. DEGs related to *SMC4* were mainly involved in pathways in cancer, PI3K-Akt signaling pathway and cell cycle pathway, which indicated that in addition to immune infiltration, there was more pathway associated with *SMC4* to explore. According to the results from our study, *SMC4* has a potential to serve as a biomarker for the evaluation of the immune cell infiltration and prognosis of sarcoma.

Several limitations of this study are worth mentioning. First, our study results with respect to the role of *SMC4* in tumors were based on the data from several databases and we only performed immunohistochemical verification on the expression level of *SMC4* in synovial sarcoma. Second, the number of patients in the database was small, and the results could be due to chance. Third, no experiments were conducted to confirm the role of *SMC4* in the development of sarcoma, and its relationship with the level of immune cell infiltration. Fourth, association of *SMC4* expression with the immune infiltration levels was weak, though it was statistically significant. This part should be interpreted with caution.

## CONCLUSION

This study showed an important association between *SMC4* and the prognosis of sarcoma and other cancers. Therefore, *SMC4* may be a useful marker to predict the survival rate and clinical consequences in patients with these cancers. The relationship of *SMC4* with immune cell infiltration was analyzed in sarcoma, which indicated that *SMC4* expression was associated with the infiltration status of immune cells in sarcoma. In summary, our study suggested that *SMC4* is related to the immune cell infiltration in sarcoma tissues and can predict the prognosis of pan-cancer. Therefore, *SMC4* has the potential to serve as a biomarker for the evaluation of the immune cell infiltration and prognosis of sarcoma.

## METHODS

### Expression levels of *SMC4* in pan-cancers in Oncomine

Expression levels of *SMC4* in different cancers were explored using Oncomine database (http://www.oncomine.org/) (Oncomine ended its service on January 17, 2022), under the setting as a *P*-value of 0.05, fold change of 2, gene rank of top 10% [27].

### Immunohistochemistry

3-μm sections of synovial sarcoma were incubated with antibodies against *SMC4* (1/100 dilution and overnight at 4°C). Then, we conjugated the sections with horseradish peroxidase antibody at 25°C for 2 hours. Thereafter, DAB (Vector Laboratories, Burlingame, CA, USA) was used to cover, and Vectashield mounting medium (Vector Laboratories) was used to mount slides. Finally, we observed all fields using light microscopy.

### Survival analysis

The relationship between *SMC4* and clinical outcome in pan-cancers was detected by GEPIA dataset (http://gepia.cancer-pku.cn/) and Kaplan-Meier Plotter databases(https://kmplot.com/analysis/) database [28]. In our study, we used GEPIA database to explore the different OS and DFS of 33 different types of cancer with high or low expression of *SMC4*. We can obtain the relationship between gene expression and the prognosis of cancer from the Kaplan-Meier Plotter database [29]. Among them, we analyzed the relationship between the expression level of *SMC4* and the prognosis of sarcoma, gastric cancer, breast cancer, liver cancer, and ovarian cancer.

### *SMC4* and immune cells infiltration

We analyzed the association between *SMC4* and tumor immune cell infiltration through the GEPIA and TIMER databases. TIMER database contains the data of tumor-infiltrating immune cells in more than 10000 samples of 32 types of cancers from TCGA [30]. Using the TIMER database, we explored the association between the *SMC4* and the infiltration level of multiple immune cells. The X-axis represents the expression level of *SMC4*, and the Y-axis represents the expression level of these marker genes [26]. In GEPIA, we also analyzed the correlation between the expression of *SMC4* and the expression of immune cell marker genes.

### GO and KEGG analysis

We obtained the gene expression profile of sarcoma from TCGA. The DEGs between the *SMC4* high group and the *SMC4* low group were analyzed using R software 4.1.2 with a threshold as |log2FC| >1 and *p* < 0.05. The GO and KEGG analysis were conducted using functional annotations from the DAVID database. GO analysis can be divided into three parts, including BP, CC and MF.

### Statistical analysis

The expression data of *SMC4* from the Oncomine database were analyzed with the *P*-value, fold change, rank and data type. The survival curve was produced by GEPIA and the Kaplan-Meier Plotter. The HR and log-rank *P* values in Kaplan-Meier plotter and GEPIA were calculated by log-rank test to compare survival curves. The correlation of gene expression in the TIMER and GEPIA databases was evaluated using Spearman correlation analysis. *P* < 0.05 was considered statistically significant [31].

### Availability of data and materials

All data generated or analysed during this study are included in this published article.

## Supplementary Materials

Supplementary Table 1
