# Supplementary material for: Association of SMC4 with prognosis and immune infiltration of sarcoma
Source: Aging (Albany NY). 2023 Jan 30;15(2):567–82. doi: 10.18632/aging.204503 (PMC9925680; doi:10.18632/aging.204503)
Supplement: Supplementary Table 1 [file aging-15-204503-s001.pdf]

SUPPLEMENTARY TABLE

Supplementary Table 1. SMC4 expression in cancers versus normal tissue in oncomine database.

Cancer	Cancer type	*P*-value	Fold change	Rank (%)	Sample	References (PMID)
Breast	Ductal Breast Carcinoma	4.87E-17	3.965	1%	47	16473279
	Medullary Breast Carcinoma	5.20E-16	2.646	1%	2136	22522925
	Invasive Ductal Breast Carcinoma	1.51E-8	2.594	3%	64	15034139
	Lobular Breast Carcinoma	5.07E-6	2.095	3%	64	15034139
Sarcoma	Leiomyosarcoma	3.48E-16	5.750	1%	158	20601955
	Pleomorphic Liposarcoma	6.20E-14	4.898	1%	158	20601955
	Myxofibrosarcoma	6.18E-16	4.866	1%	158	20601955
	Dedifferentiated Liposarcoma	8.36E-15	3.671	1%	158	20601955
	Myxoid/Round Cell Liposarcoma	1.52E-7	2.839	8%	158	20601955
	Fibrosarcoma	7.26E-9	8.252	1%	54	15994966
	Leiomyosarcoma	1.32E-8	8.448	1%	54	15994966
	Pleomorphic Liposarcoma	4.78E-8	6.595	1%	54	15994966
	Malignant Fibrous Histiocytoma	5.78E-9	8.103	1%	54	15994966
Bladder	Infiltrating Bladder Urothelial Carcinoma	7.59E-9	3.479	1%	60	15173019
	Superficial Bladder Cancer	3.69E-8	2.670	2%	60	15173019
Liver	Hepatocellular Carcinoma	5.37E-13	4.138	1%	43	21159642
	Hepatocellular Carcinoma	4.91E-87	3.747	1%	445	21159642
	Hepatocellular Carcinoma	1.84E-13	2.010	3%	197	12058060
Renal	Clear Cell Renal Cell Carcinoma	5.72E-11	2.767	1%	20	17699851
	Non-Hereditary Clear Cell Renal Cell Carcinoma	6.30E-12	2.606	1%	70	19470766
	Hereditary Clear Cell Renal Cell Carcinoma	1.22E-13	2.529	1%	70	19470766
	Renal Pelvis Urothelial Carcinoma	2.00E-5	2.705	10%	92	16115910
Lung	Squamous Cell Lung Carcinoma	1.60E-9	19.160	1%	203	11707567
	Small Cell Lung Carcinoma	2.36E-7	9.650	1%	203	11707567
	Squamous Cell Lung Carcinoma	1.95E-12	2.347	1%	93	15833835
	Tongue Squamous Cell Carcinoma	4.94E-11	2.002	2%	93	15833835
	Squamous Cell Lung Carcinoma	5.70E-11	2.533	4%	156	20421987
	Large Cell Lung Carcinoma	1.93E-6	2.464	5%	156	20421987
Colon	Rectal Adenoma	6.98E-12	2.195	1%	64	18171984
	Colon Adenoma	5.76E-10	2.022	8%	64	18171984
	Colon Carcinoma	1.67E-7	2.146	6%	40	20957034
	Colon Adenoma	6.15E-5	2.602	9%	40	20957034
Cervix	Cervical Squamous Cell Carcinoma Epithelia	1.74E-10	3.312	1%	41	17974957
	Cervical Squamous Cell Carcinoma	1.55E-10	3.384	1%	66	18506748
	Cervical Squamous Cell Carcinoma	1.51E-6	3.305	6%	45	18191186
Ovarian	Ovarian Serous Adenocarcinoma	5.67E-8	2.773	1%	50	15161682
Vulva	Vulvar Intraepithelial Neoplasia	1.16E-7	3.712	1%	19	17471573
Head-Neck	Oral Cavity Squamous Cell Carcinoma Epithelia	7.00E-6	2.461	1%	20	15381369
	Nasopharyngeal Carcinoma	7.42E-7	2.476	2%	41	16912175
	Tongue Squamous Cell Carcinoma	7.09E-7	2.328	8%	58	19138406
Brain	Anaplastic Oligoastrocytoma	6.01E-5	6.826	1%	33	16357140
	Anaplastic Oligodendroglioma	2.22E-7	4.220	2%	33	16357140
	Glioblastoma	9.84E-21	3.695	1%	180	16616334
	Anaplastic Astrocytoma	1.39E-5	2.514	7%	180	16616334

	Glioblastoma	9.63E-7	4.010	4%	54	16204036
	Glioblastoma	2.00E-6	6.163	5%	84	18565887
Blood	Plasma Cell Leukemia	7.03E-5	3.045	3%	158	19396863
Gastric	Gastric Mixed Adenocarcinoma	2.27E-6	2.293	2%	69	19081245
	Gastric Intestinal Type Adenocarcinoma	1.47E-10	2.377	3%	69	19081245
Skin	Skin Squamous Cell Carcinoma	3.05E-5	2.237	3%	87	18442402
Lymph	Unspecified Peripheral T-Cell Lymphoma	3.78E-11	2.441	5%	60	17304354
	Angioimmunoblastic T-Cell Lymphoma	1.70E-5	2.412	7%	60	17304354
